# Impact of climate extreme events and their causality on maize yield in South Africa

**DOI:** 10.1038/s41598-023-38921-0

**Published:** 2023-08-01

**Authors:** Christian Simanjuntak, Thomas Gaiser, Hella Ellen Ahrends, Andrej Ceglar, Manmeet Singh, Frank Ewert, Amit Kumar Srivastava

**Affiliations:** 1 Institute of Crop Science and Resource Conservation, University of Bonn, Katzenburgweg 5, 53115 Bonn, Germany; 2 Department of Agricultural Sciences, University of Helsinki, Koetilantie 5, 00014 Helsinki, Finland; 3 Climate Change Centre of the European Central Bank, Sonnemannstrasse 20, 60314 Frankfurt Am Main, Germany; 4 Indian Institute of Tropical Meteorology, Ministry of Earth Sciences, Pune, India; 5 Leibniz Centre for Agricultural Landscape Research (ZALF), Eberswalder Straße 84, 15374 Müncheberg, Germany

**Keywords:** Climate and Earth system modelling, Climate-change impacts, Climate-change mitigation

## Abstract

Extreme climate events can have a significant negative impact on maize productivity, resulting in food scarcity and socioeconomic losses. Thus, quantifying their effect is needed for developing future adaptation and mitigation strategies, especially for countries relying on maize as a staple crop, such as South Africa. While several studies have analyzed the impact of climate extremes on maize yields in South Africa, little is known on the quantitative contribution of combined extreme events to maize yield variability and the causality link of extreme events. This study uses existing stress indices to investigate temporal and spatial patterns of heatwaves, drought, and extreme precipitation during maize growing season between 1986/87 and 2015/16 for South Africa provinces and at national level and quantifies their contribution to yield variability. A causal discovery algorithm was applied to investigate the causal relationship among extreme events. At the province and national levels, heatwaves and extreme precipitation showed no significant trend. However, drought severity increased in several provinces. The modified Combined Stress Index (CSIm) model showed that the maize yield nationwide was associated with drought events (explaining 25% of maize yield variability). Heatwaves has significant influence on maize yield variability (35%) in Free State. In North West province, the maize yield variability (46%) was sensitive to the combination of drought and extreme precipitation. The causal analysis suggests that the occurrence of heatwaves intensified drought, while a causal link between heatwaves and extreme precipitation was not detected. The presented findings provide a deeper insight into the sensitivity of yield data to climate extremes and serve as a basis for future studies on maize yield anomalies.

## Introduction

Extreme climate and weather events could threaten crop productivity and trigger food insecurity globally. Aside from natural variability, the main driver of unpredictable frequency, intensity, spatial extent, and duration of such events is climate change^1^. In the long term, this may lead to widespread hunger across the globe, especially under consideration of a growing population that is expected to reach 8.5 and 9.7 billion in 2030 and 2050, respectively^2^. Therefore, improving our understanding of climate extremes impact on crop productivity will enhance our adaptation towards climate change.

Maize is one of the most important crops and a major food source (> 30% calories) for about 4.5 billion people spread over 94 developing countries^3^. According to FAOSTAT 2019^4^, the total area of maize harvested worldwide is around 195 million ha with total production reaching 1138 megatonnes. In 2030, maize acreage is expected to increase to 227 million, overtaking wheat acreage^5^. This is largely due to meet the increasing demand; indeed, maize is needed not only for food consumption but also for livestock feed and bioethanol production^6,7^.

For South Africa, as the highest maize producer in the African continent, maize becomes a staple food and contributes to the economy of the country^8^. Large-scale commercial producers dominate maize production; however, smallholder farmers mainly engage with maize production to support their socio-economic livelihoods^9,10^. FAOSTAT reported that South Africa produced 11.2 million tonnes of maize in 2019 within an area harvested of 2.3 million hectares^4^. The provinces of Free State, KwaZulu-Natal, Mpumalanga, and North West accounted for 43%, 4.5%, 24%, and 16% of the total national maize production in the 2020/2021 season (data provided by the Statistics and Economic Analysis of Agriculture, Forestry and Fisheries Department of South Africa). Partially maize is exported to neighboring countries (3 million tonnes in the 2021/2022 season) but most are for local consumption^11^. Given the importance of maize production for local supply, extreme climate anomalies (heatwaves, drought, extreme precipitation) could threaten the national food security of South Africa^12^.

Heatwaves, describing a prolonged period of high temperature above certain temperature thresholds, could inhibit maize development^13^. Exposure to high temperatures during the maize reproductive stage will affect pollen viability, fertilization, and grain development, which can significantly reduce maize yield between 80 and 90%^14–17^. Begcy et al.^18^ reported that after having been exposed 48 h to heat stress, maize pollen damage resulted in low pollen germination rates which led to low yields. At the same time, the intensity of heatwaves could amplify drought severity which is associated with a soil moisture deficit, thereby negatively affecting plants water fluxes^19^.

A recently suggested method to measure the intensity of heatwaves had been proposed by Russo et al.^20^, which is known as Heat Wave Magnitude Index daily (HWMId). It is calculated based on the maximum magnitude heatwave in a year. In order to account for the importance of heat events during the growing season of the crop, Zampieri et al.^21^ suggested the Heat Magnitude Day (HMD), which is calculated based on the critical plant phenological stages prior to harvest. Zampieri et al.^22^ and Ceglar et al.^23^ successfully applied the HMD to investigate the variation of maize production on a global scale and maize yield at the national level (Europe countries).

The combination between increasing potential evapotranspiration and a prolonged period of high temperature associated with low precipitation will lead to a drought event^24^. A model-based study by Whan et al.^25^ highlighted that a decrease in soil moisture by about 100 mm is associated with an increase in monthly maximum temperatures of 1.6 °C in Southern Central and Southeastern Europe. Overall, there is a negative impact of drought on crop yields, such as for barley, rice, and wheat^26–28^. For maize, decreasing leaf number, abnormal root formation, slow growth rate, and lower chlorophyll content are common responses to drought stress^29–31^. In China, severe drought reduced maize yield by up to 14%^32^.

To evaluate and quantify the intensity of drought events, the standardized evapotranspiration index (SPEI) and the standardized precipitation index (SPI) are commonly applied. While the SPI only considers precipitation amounts, the SPEI takes into account the water balance between precipitation and temperature in the form of evapotranspiration^33^. In the context of global warming, the increasing temperature increases drought severity due to the increased atmospheric demand, highlighting the importance to consider temperature effects^34^. It had been shown that the SPEI shown a consistent performance in drought monitoring in Pakistan compared to 15 various drought indices^35^. In a recent study by Omolola et al.^36^ for 4 major maize producing South African provinces (identical to the study area used in this study) discovered that the SPEI-3, calculated based on meteorological data from 27 ground stations, was significantly correlated with maize yield during the growing season from 1990 to 2015. Supported by this result, this study adopts drought analysis based on SPEI 3-month time scales.

Under a rising global temperature, the intensity of global mean precipitation can be significantly increased, due to changes in precipitable water content in the atmosphere (Clausius Clapeyron relationship)^37–39^ and energy budget^40^. It is predicted that under future global warming the magnitude of extreme precipitation is likely to get stronger^41^. The impact of extreme precipitation on crop production can be devastating. Excessive rainfall is not only damaging a plant by initiating fungal and bacterial diseases but also creating anaerobic conditions (waterlogging), leading to nutrient loss and soil erosion^42–44^. In the United States, excessive rainfall reduced maize yields by up to 34%^45^.

Some indices have been proposed to quantify extreme precipitation with reference to the intensity and the frequency, for instance: maximum annual 1- or 5-day precipitation, precipitation above percentile thresholds, and the number of consecutive wet days^46,47^. Among these methods, the percentile threshold has been widely applied to calculate the intensity of extreme precipitation using both observations and modeled data^41,48,49^.

The negative impact of climate extremes on maize yields in South Africa has been assessed in several studies, which are often based on crop and/or climate model simulations (e.g., Mangani et al.^50^, Bradshaw et al.^12^), Existing studies mostly focus on future extreme scenarios and past trends of climate data in their absolute or relative impact on crop yields. However, to the best of our knowledge, the contribution of the different climate extreme events (heatwaves, drought, and extreme precipitation) to maize yield variability, based on climate data tailored to the crop growing season for the different provinces in South Africa has not been quantified yet. Further, cited studies do not evaluate the causal relation among extreme events in more detail. Thus, little is known about the causal network among them, i.e., among indices that are commonly used to quantify these events. By now, novel statistical data analysis techniques, such as Peter and Clark Momentary Conditional Independence (PCMCI) allow for assessing such relations^51^, thereby providing a deeper understanding of causal networks of these climate extreme events during maize growing season.

Not many studies have applied the combination of extreme events analysis in investigating maize yield variability. Such an index known as the Combined Stress Index (CSI) was originally formulated based on a linear combination of indices describing heatwave and drought events^21,23^. To understand the impact of excess water events in South Africa, the extreme precipitation index is added to the equation. This study aims to fill these existing knowledge gaps using data from South Africa at the province level. Thus, the objectives of this study are (1) investigating the characteristics of climate extremes such as heatwaves, drought, and extreme precipitation during the maize growing season, (2) quantifying the contribution of these extreme events to maize yield variability, and (3) assessing the causal relationship among these events.

## Materials and methods

### Data collection

**Figure 1:**
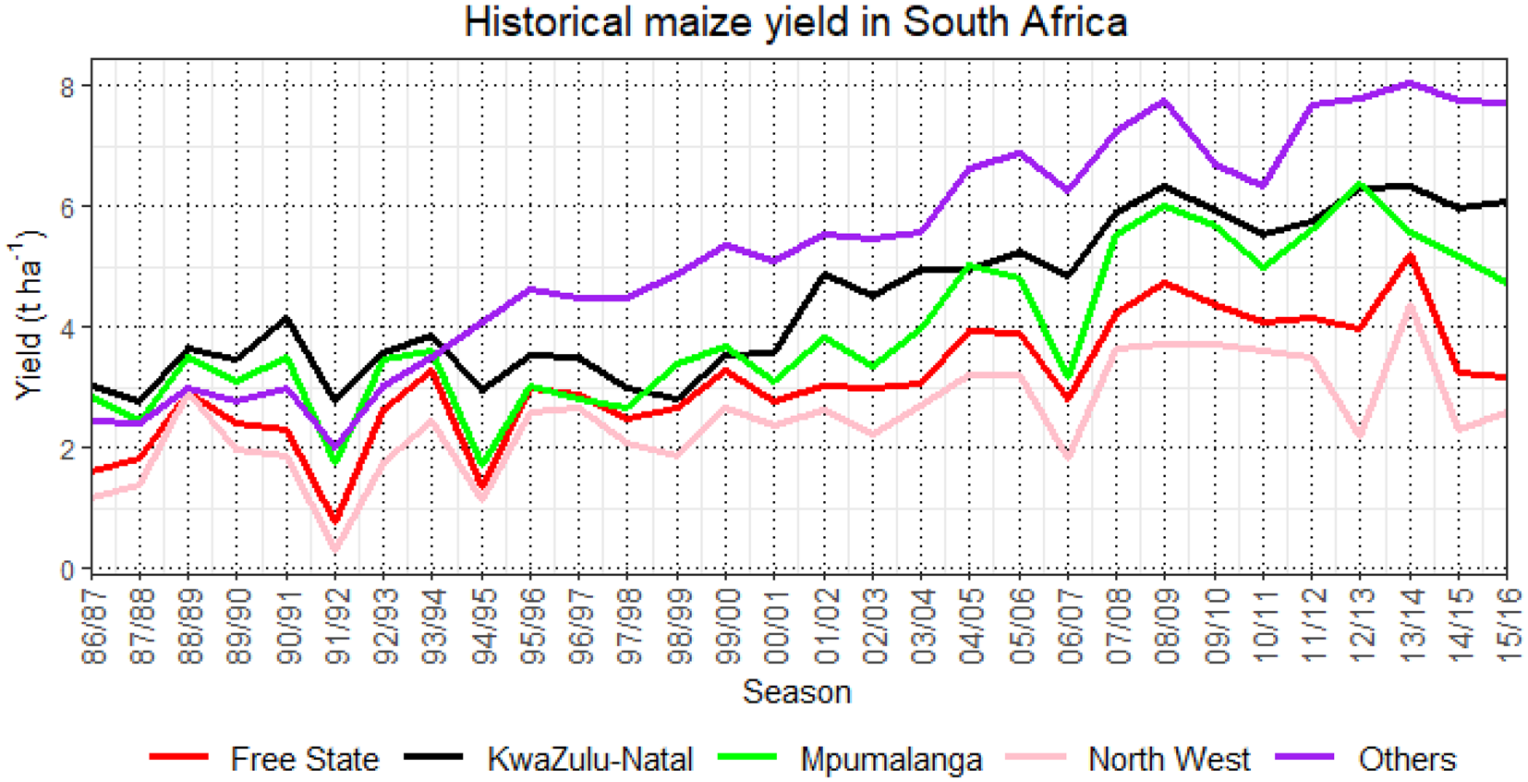
Historical maize yield (t ha^−1^) in South Africa for the different provinces between the season of 1986/87 and 2015/16.

Historical maize yield data of South Africa from production season 1986/87–2015/16 was provided directly by the Statistics and Economic Analysis of Agriculture, Forestry and Fisheries Department of South Africa (Fig. 1). The complete maize yield data do not distinguish irrigated and rainfed maize production. The majority of maize production in South Africa is produced under rainfed condition^50,52^. Only 10% of arable land is being used under irrigation scheme from approximately 1.3 million hectares^53^. According to South Africa National Land Cover (SANLC), the percentage distribution land between irrigated and rainfed cropland are 0.81% and 7.61%, respectively (Fig. S1)^54^. Therefore, it can be assumed that the historical maize yield data is based on rainfed conditions.

The maximum daily temperature (T_max_ in Celsius) and precipitation flux (P_flux_ in mm day^−1^) were retrieved from the reanalysis AgERA5 dataset which is a global gridded data set developed for agro-ecological models and applications in agriculture-related research^55^. It is available at 0.1° × 0.1° resolution from 1979 until the present. In this study, T_max_ and P_flux_ data from 1986/87 up to 2015/16 were extracted and further used as a study period to quantify the magnitude of heatwaves and extreme precipitation. The drought indicator was retrieved from the Global SPEI (Standardized Precipitation Evapotranspiration Index) database (https://spei.csic.es/index.html) which is available as global gridded monthly time series data with a resolution of 0.5° spanning from 1901 to 2015 (version 2.5)^56^.

The study area was divided into Free State, KwaZulu-Natal, Mpumalanga, and North West as major maize production provinces. In addition, the rest of the South African provinces, Northern Cape, Western Cape, Eastern Cape, Gauteng, and Limpopo, were considered as “Others” regions. This was motivated by their lower contribution to the total maize production. We also integrated all regions which were identified as “South Africa” (Fig. S2).

### Definition of climate extreme events and data processing

Using data from a single country and working at the province scale, heatwave was defined as at least 3 consecutive days with maximum temperatures (T_max_) above the daily threshold^57^. The daily threshold was calculated as the 90th percentile of the daily temperature maximum centered on a 31 days window (T_90_)^20,58,59^. Therefore, the threshold for a particular day (*d*) was defined from the 90th percentile of the data set (A*d*),

1 $$\mathrm{A}d=\bigcup_{s=1986/87}^{2015/16}\bigcup_{i=d-15}^{d+15}{T}_{s,i},$$

where U represents the union of maximum temperature data sets and T_s,i_ indicates the daily T_max_ in a particular day *i* in the season s.

In this study, the heatwave magnitude index daily (HWMId) was adjusted to account for the maize growing season and phenology as described by Zampieri et al.^22^. It was denoted as Heat Magnitude Day (HMD)^21^. Because the planting date varies among the regions in South Africa, maize was expected to mature in March. In order to capture the impact of heatwave events during the critical period of maize development before harvest, the cumulative HMD within a 5-month time window counting backward one month before harvesting was computed. Thus, it started in October and ended in February of the following year^52,60–62^.

Based on HWMId, the daily magnitude heatwave (M_d_) was calculated as follows:

2 $${M}_{d}\left({T}_{d}\right)=\left\{\begin{array}{ll}\frac{({T}_{d}-{T}_{30y25p})}{({T}_{30y75p}-{T}_{30y25p})} & \quad if\; {T}_{d}>{T}_{90}\\ 0 & \quad if \; {T}_{d}\le {T}_{90}\end{array}\right.$$

where T_d_ denotes as the daily maximum temperature of day d. T_30y25p_ and T_30y75p_ represent the 25th and 75th percentile temperatures during the study period (30 years range). The attribute of HMD frequency was determined as the total number of days (at least 3 consecutive days) above the daily threshold (T_90_) for each growing season^63^.

The Standardized Precipitation Evapotranspiration Index (SPEI) (see “Data collection” section) was applied for investigating the impact of soil moisture anomalies on maize yield. The SPEI considers the effect of potential evapotranspiration (PET) using the FAO-56 Penman–Monteith equation, thereby representing a simple climatic water balance. In this study, we used the 3-month time scale SPEI data set (SPEI-3)^36^. The time window was set in parallel with the HMD time window (5-months) to capture the yield variability due to drought stress. The severity of dry spell increased with the decreased of SPEI value, in the opposite direction, the high SPEI value indicated wet conditions^64^.

The extreme precipitation amount was defined as daily precipitation (*PP*_*d*_) that exceeds the 95th percentile (*P95th*) of the study period^41,49,65^. The time window data differed with that used for HMD and SPEI: the complete 6-month time period from October to March of the following year was used in order to capture the negative impact of excessive precipitation events during maturity^66,67^. The frequency of extreme precipitation was defined when the days have precipitation above the 95-percentile threshold. To quantify the magnitude of extreme precipitation during the maize growing season, the total precipitation of daily mean precipitation (d) which exceeded the 95th percentile threshold during each season was computed. It is described as Extreme Precipitation Modified (EPM) given by:

3 $$EPM=\sum \limits_{d=1}^{n}{PP}_{d}, which {PP}_{d}> {P95}{th}$$

### Trend analysis

To identify and quantify temporal trends in climate extremes (HMD, SPEI, and EPM), we employed the Mann–Kendall test and Sens’s slope, respectively, for each region (cf. Fig. S2 for provinces). Autocorrelation for trend analysis was performed using autocorrelation function (ACF) and partial autocorrelation (PACF). These tests revealed no significant serial correlation. Linear regression was applied to recognize the trend tendency of climate extremes. Furthermore, the spatial distribution of their magnitudes during the observation period was shown. The maps presented in this manuscript were generated using QGIS 3.16.7-Hannover software, https://www.qgis.org/en/.

### Relation between extreme events and maize yield: Combined Stress Index

To assess the overall influence of extreme climate events on maize yield in South Africa, we applied the adjusted version of the Combined Stress Index (CSI) developed by Zampieri et al.^21^ and Ceglar et al.^23^. The CSI is formulated based on a linear combination of indices describing heatwave and drought events. In this study, an additional predictor related to extreme precipitation events was added to the equation.

Because long-term trends in crop yield can be affected by improvements in agriculture practices, the observed yield was detrended by subtracting the locally estimated scatterplot smoothing (LOESS method)^68^ where the span was determined by fivefold cross-validation. The span that has the lowest Mean Squared Error (MSE) was selected. In this way, climate extreme effects (restricted to events during the growing season) on maize yield anomalies could be isolated. The detrending of extreme indices using LOESS method was also performed when the data demonstrated a significant trend (*Mann–Kendall*) or breaks points (*strucchange*), in the opposite case, the mean was removed. The modified Combined Stress Index (CSIm) was formulated by a linear combination of HMD, SPEI, and EPM (cf. “Definition of climate extreme events and data processing” section) using the following equation:

4 $$CSIm=\alpha .{HMD}_{detrend,s}^{std}+\beta .{SPEI}_{detrend,s}+ \gamma .{EPM}_{detrend,s}^{std}+ {\varepsilon }_{s}$$

with *s* as a maize growing season and *std* indicating a standardized values. SPEI was only detrended as it is standardized by definition^23^. Prior to analysis, the multicollinearity of explanatory variables was assessed by identifying tolerance value/variance inflation (VIF) and condition index^69^. Our analysis showed that the VIF of explanatory variables was not above 5 to 10 and the tolerance was not lower than 0.1 to 0.2 (Table S1). Furthermore, the condition index of explanatory variables was not larger than 10 (Table S2)^70^. This indicated that intercorrelated variables did not exist. Thus, the coefficients α, β, and γ were calculated by multiple linear regression of indices values on observed yield anomalies at the regional level^21,22^.

### Relation among climate extremes: Gaussian process regression and causality analysis

The Gaussian process regression was carried out to test the non-linear dependency among HMD, SPEI, and EPM for causality^71^. The magnitude of each index (cf. “Definition of climate extreme events and data processing” section) were analyzed based on maize growing season. Gaussian Process Regression (GPR) is a type of Bayesian non-parametric regression. It is a probabilistic model that works well for both regression and classification^72^. To estimate the noise level of data, a standard radial basis function kernel including a white kernel was applied. Furthermore, the hyperparameters of the kernel was optimized using log-marginal-likelihood (LML).

In order to understand the complex data-driven causal relationships between HMD, SPEI, and EPM, the Peters and Clark Momentary Conditional Independence (PCMCI) were performed. Peters and Clark Momentary Conditional Independence (PCMCI), is a causal discovery method for determining what factors in a dataset are causally related to one another^51^. This technique has been developed for handling high dimensional data sets with time-dependent and non-linear relationships when aiming to estimate causal networks. It relies on the concept of temporary conditional independence, which holds that if two variables are causally coupled, then the inclusion of a third variable should not alter their mutual reliance. First, we identified a collection of potential explanatory factors to use the MCI method. Next, the algorithm employed a technique to check for temporary conditional independence between pairs of these variables. To do this, we estimated the mutual information between each pair of variables and then checked to see if it was substantially different from zero after adjusting for the other variables in the dataset. The existence of a causal link between two variables was shown by a non-zero value for the mutual information between them when all other variables were held constant. In this case, the direction of the mutual information indicates the direction of the causal link, with the variable that has greater mutual information being regarded as the cause and the variable that has lower mutual information being considered the consequence. A causal graph, where the edges reflect the associations between variables, can be constructed once all possible pairings of variables have been evaluated for momentary conditional independence. We used the “tigramite” library developed for Phyton Programming Language (Phyton Software Foundation. Phyton Language Reference, version 5.2 available at https://github.com/jakobrunge/tigramite)^51^.

## Results

**Figure 2:**
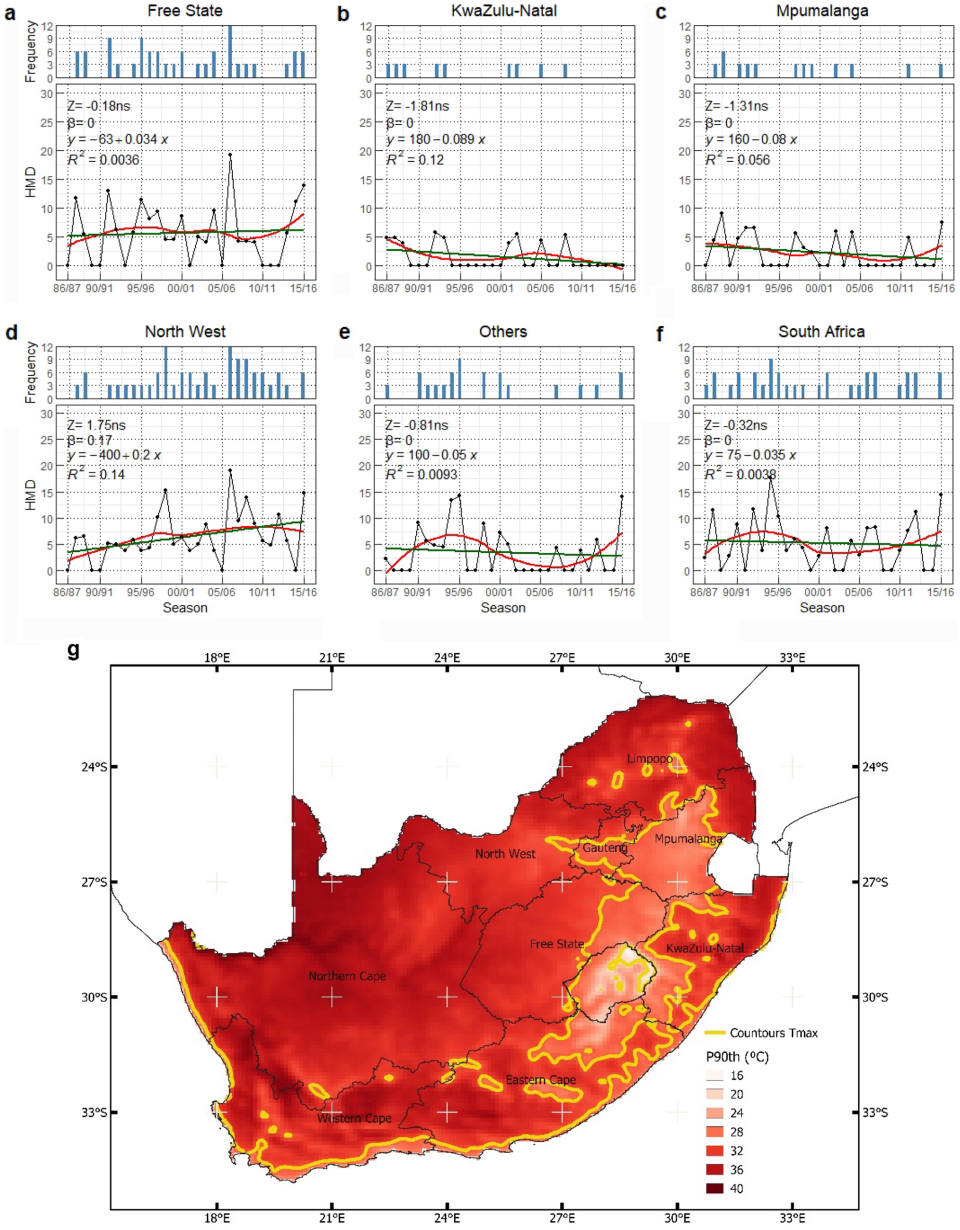
The trend (Z), the magnitude (β), and the corresponding frequency of Heat Magnitude Day (HMD) seasonally for (**a**) Free State, (**b**) KwaZulu-Natal, (**c**) Mpumalanga, (**d**) North West, (**e**) Others, and (**f**) South Africa, including (**g**) the spatial patterns of the 90th percentile of daily maximum temperatures (Tmax) from 1986/87 to 2015/16 maize growing season. The red line indicates the non-linear trend (LOESS) of heatwave magnitude. The linear regression lines for the inclination of sloping are shown in green. *ns,* symbolize no significant trend. The yellow contour lines on the map distinguish the spatial patterns.

**Figure 3:**
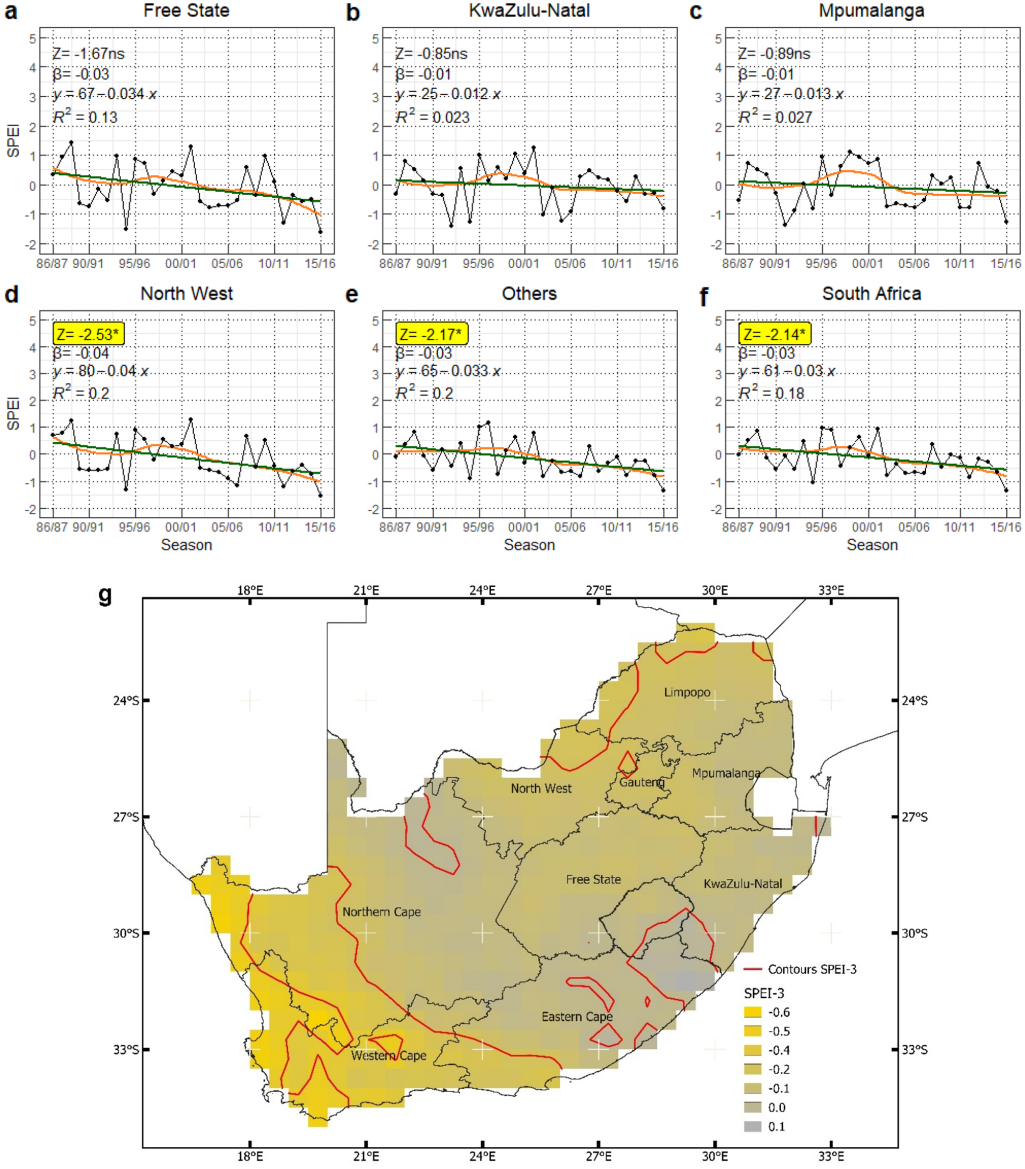
The trend (Z) and the magnitude (β) of Standardized Precipitation Evapotranspiration Index (SPEI) seasonally for (**a**) Free State, (**b**) KwaZulu-Natal, (**c**) Mpumalanga, (**d**) North West, (**e**) Others, and (**f**) South Africa, including (**g**) the spatial patterns of mean SPEI-3 during maize growing season from 1986/87 to 2015/16. The orange lines indicate the non-linear trend (LOESS) of the drought index. The linear regression lines for the inclination of sloping are shown in green. *ns,* symbolize no significant trend. (*) denotes p-value ≤ 0.05. The red contour lines on the map distinguish the spatial patterns.

**Figure 4:**
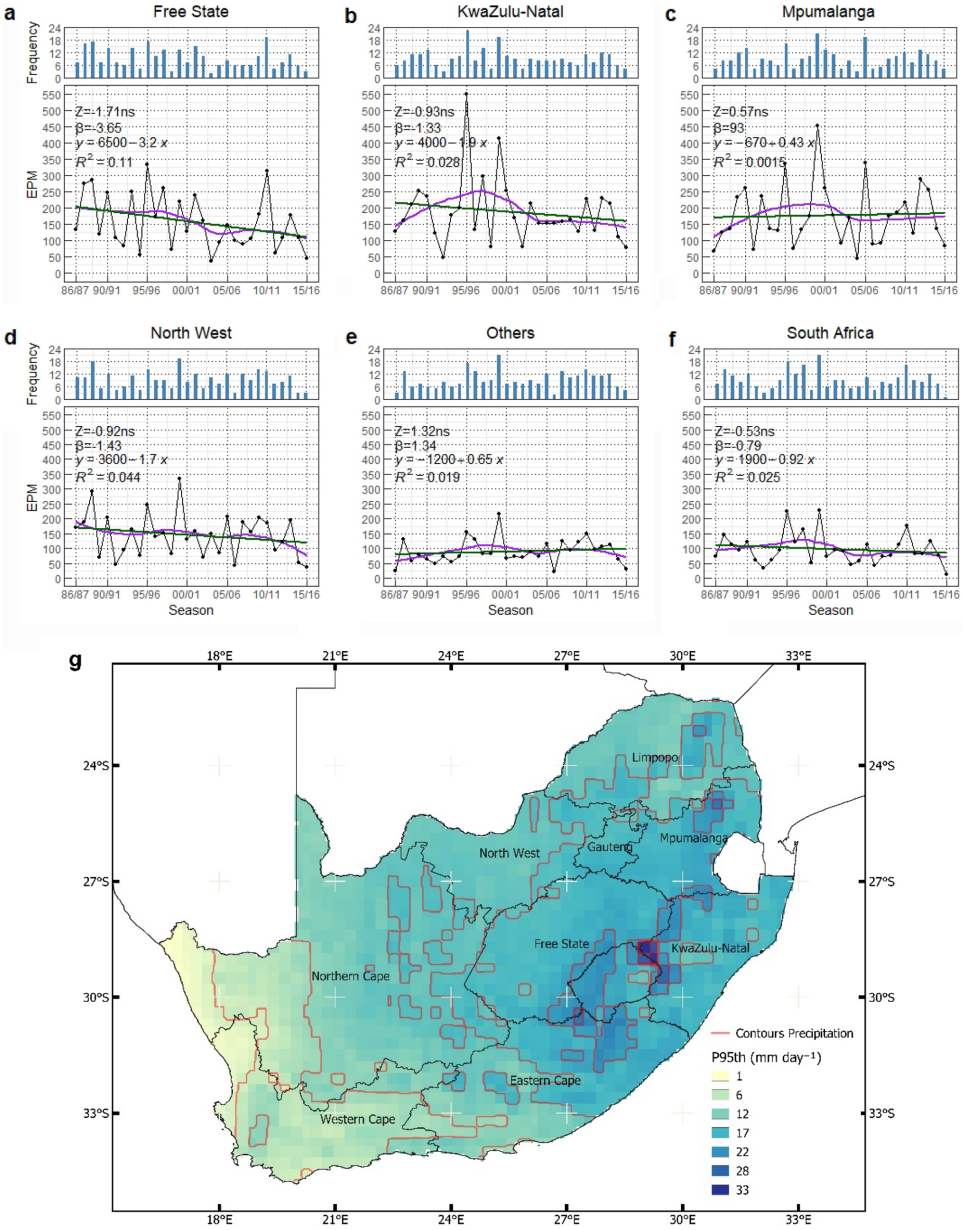
The trend (Z), the magnitude (β), and the corresponding frequency of Extreme Precipitation Modification (EPM) seasonally for (**a**) Free State, (**b**) KwaZulu-Natal, (**c**) Mpumalanga, (**d**) North West, (**e**) Others, and (**f**) South Africa, including (**g**) the 95th percentile of daily precipitation during maize growing season from 1986/87 to 2015/16. The purple line indicates the non-linear trend (LOESS) of the extreme precipitation index. The linear regression lines for the inclination of sloping are shown in green. *ns,* symbolizes no significant trend. The orange contour lines on the map distinguish the spatial patterns.

This study assessed individual and the combined effect of heat, drought, and extreme precipitation events (Figs. 2, 3, and 4) on maize yield anomalies in South Africa. The combined effects were quantified using a modified version of the Combined Stress Index (CSIm).

Figure 2 shows the temporal pattern and the occurrence of heat magnitude day (HMD), as an indicator for heat stress events. There were no significant trends of HMD in Free State province during maize growing season for the last 30 years. The highest magnitude at 19 HMD in 2006/07 season shows the highest frequency of extreme heatwaves at 12 (Fig. 2a). This event similarly occurred in North West province (Fig. 2d). Comparing the HMD between 1998/99 and 2006/07, respectively, which had similar frequency at 12, demonstrated that North West province experienced less intense heatwaves in the 1998/99 growing season at 15 HMD. The KwaZulu-Natal province shows the absence of heatwave between season 1994/95–2000/01, and continue to flatten after 2009/10 season (Fig. 2b). Likewise in Mpumalanga province, there was a consecutive absence of heatwave between the 2005/06–2010/11 growing season (Fig. 2c). For Others regions, multiple peaks of HDMI were identified in 1994/95, 1995/96, and 2015/16 seasons (Fig. 2e). The HMD trend was not identified for the entire South Africa despite the fluctuation throughout the seasons (Fig. 2f). It can be seen that the spatial distribution of heatwaves based on the 90th percentile of daily maximum temperatures during the study period was more intense in the northwestern of South Africa where Free State, North West, and most Other regions are located (Fig. 2g).

Figure 3 illustrates the temporal and spatial pattern of severe drought occurrences across South Africa during maize growing seasons with decreasing SPEI values indicating stronger drought periods. We observed a decreasing level of the standardized evapotranspiration index (SPEI) in all regions. However, only North West, Others, and South Africa showed a significant downward trend with − 0.04, − 0.03, and − 0.03 SPEI per season, respectively (Fig. 3d–f). These results indicate that South Africa in general experienced an increasing number of dry spells and increasing drought intensity during maize growing seasons over time (reaching SPEI < − 1.5 in the last study period). It is worth noting that there was an increase in the SPEI (indicating lower drought severity and a moderate wet season with SPEI ranging from 0 to 1.5) between 1995/96 and 2001/02 for all study regions. For Free State and North West, the 1994/95 season was found to be the most severe drought period (SPEI < − 1) (Fig. 3a,d). Likewise, for Kwazulu-Natal and Mpumalanga, the most significant drought (SPEI of − 1.4) was recorded in the season of 1992/93 and 1991/92, respectively (Fig. 3b,c). The spatial patterns of SPEI clearly illustrate that, during the last three decades, the southwestern and northeastern regions of South Africa in particular have been affected by drought events (Fig. 3g).

The temporal patterns of extreme precipitation events and corresponding frequency showed strong fluctuations and seasonal independence for all study areas (Fig. 4). Thus, the statistically significant trends of extreme precipitation could not be identified. Each province exhibited individual patterns and ranges. In the Free State (Fig. 4a), EPM was recorded at 333 during the 1995/96 growing season at the frequency of 17. With the same frequency in the 1988/89 growing season, the magnitude of EPM was lower (287). This indicates that extreme precipitation was more severe in 1995/96. Similarly in Kwazulu-Natal (Fig. 4b), the 1995/96 growing season showed the highest degree of EPM up to 549. In the northeastern regions, such as Mpumalanga and North West (Fig. 4c,d), the severe record was identified in 1999/00 at 453 and 334 with frequency at 21 and 19 respectively. At the same time, Others provinces and the entire South Africa showed the highest amplitude of EPM. Our results further demonstrated that extreme precipitation in South Africa above the 95th percentile occurred every growing season for the last three decades. This extreme precipitation primarily affected the eastern regions of South Africa covering largely Free State, KwaZulu-Natal, Mpumalanga, and partly Eastern Cape provinces (Fig. 4g). On the other hand, the 95th precipitation percentile below 12 mm day^−1^ dominated in southwestern regions which were primarily represented by Others regions (Fig. 4g).

The modified CSI (CSIm) was employed to capture the combination effect of heat stress (α), drought (β), and excess water events (γ) on maize yield variability in South Africa (Table 1). The yield in Free State was moderately influenced by heatwaves where CSIm explained 35% of inter-sessional yield anomalies. Furthermore, for the North West, maize yield variability was rather associated with the combined effect of drought and extreme rainfall. The CSIm was able to capture 46% of the inter-sessional yield variability. There was no significant effect of extreme events on maize yield in KwaZulu-Natal, Mpumalanga, and Others provinces. Therefore, these provinces were excluded from the CSIm analysis. The CSIm also suggested that extreme heatwave did not play a significant role in influencing maize yield variability at national scale. It was rather affected by soil moisture deficits which explained 25% maize yield variability.

**Table 1:** The modified Combined Stress Index (CSIm) coefficients (α, β, γ) and coefficient of determination for each region.

Regions	α	β	γ	R^2^
Free State	− 0.41*	0.23	0.22	0.35
KwaZulu-Natal	0.38	0.13	0.23	0.17
Mpumalanga	0.09	0.38	0.27	0.20
North West	− 0.16	0.57*	0.40*	0.46
Others	0.01	− 0.04	− 0.23	0.06
South Africa	− 0.13	0.75*	0.17	0.25

*p-value ≤ 0.05.

**Figure 5:**
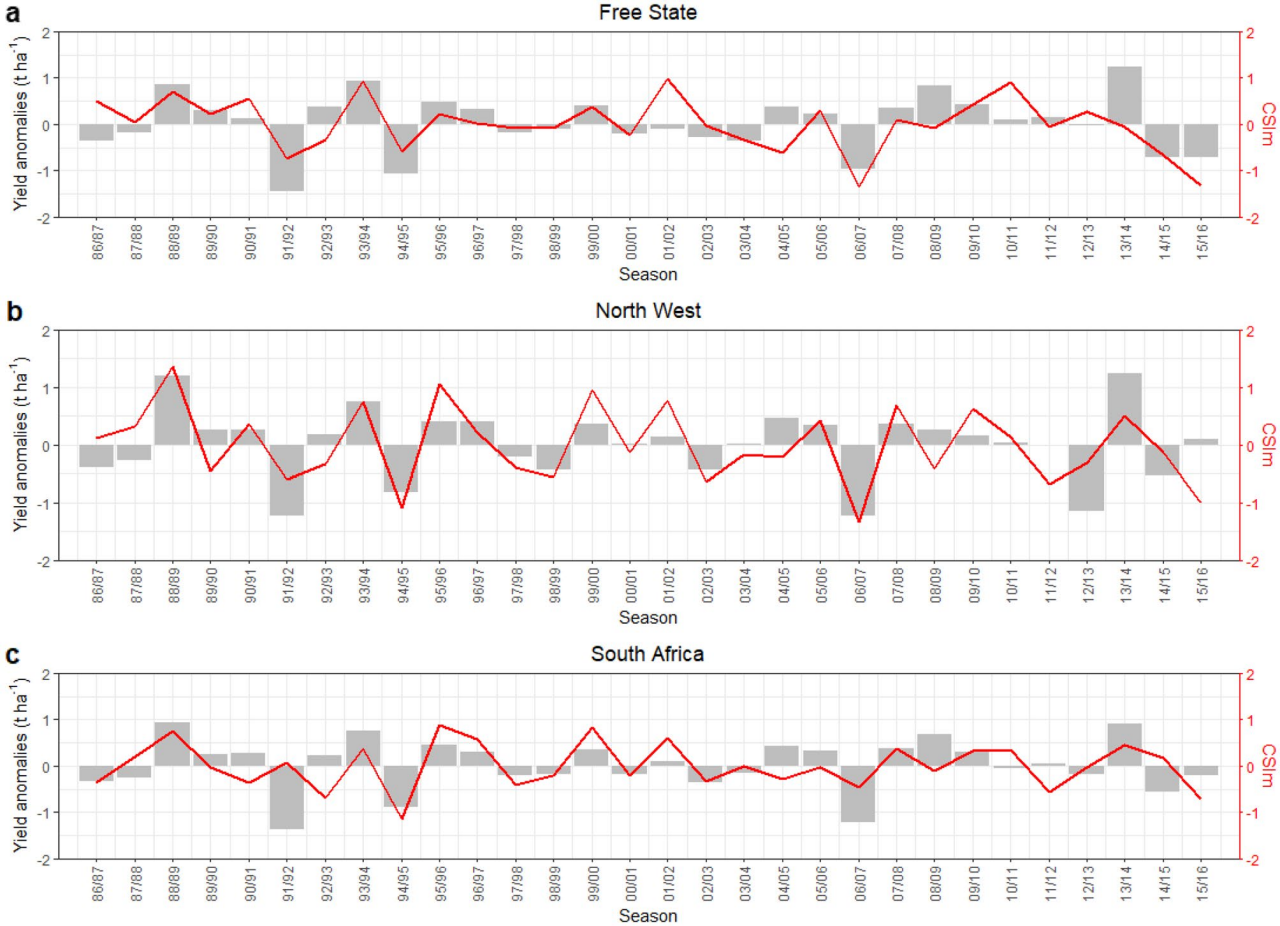
Province-based, namely: (**a**) Free State, (**b**) North West, and (**c**) National scale time-series maize yield anomalies (left-hand axis, grey bar) and Combined Stress Index (CSIm) (right-hand axis, red line) from 1986/87 to 2015/16 growing season.

The inter-seasonal fluctuations of the CSIm were similar to the pattern of maize yield anomalies (Fig. 5). These findings need to be interpreted with caution. In the growing season of 1991/92, 1994/95, 2006/07, 2014/15, and 2015/16, the CSIm was able to capture the substantial negative maize yield anomalies in the Free State (Fig. 5a). This finding shows that negative yield anomalies associated with the negative CSIm. For North West (Fig. 5b), the negative CSIm model shows negative yield losses with similar growing season with Free State except in 2015/16 growing season. In addition, 1998/99 and 2002/03 season shows noticeable negative yield anomalies. At the national scale (Fig. 5c), the season characterized by positive yield anomalies and significant high amplitude of CSIm occurred in 1988/89, 1993/94, 1995/96, 1999/00, 2007/08, and 2013/14.

**Figure 6:**
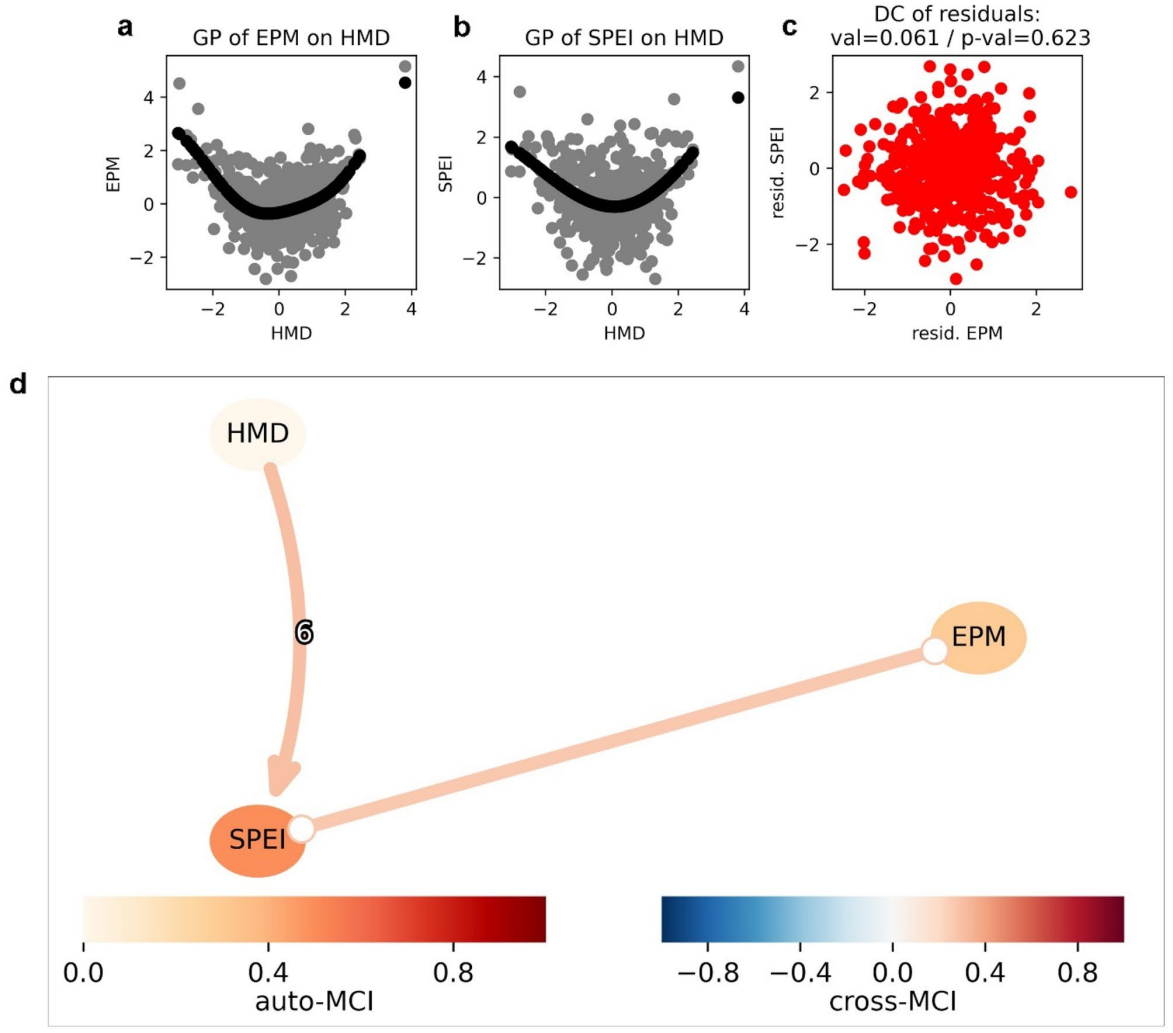
Gaussian process regression of (**a**) EPM on HMD, (**b**) SPEI on HMD, (**c**) the scatter plot of residuals of EPM and SPEI, and (**d**) Causal discovery graph generated using PCMCI showing the relationships of HMD, SPEI, and EPM including the time lags (link labels) during maize growing season from 1986/87 to 2015/16. The node colors indicate the nonlinear auto-dependency of each variable (auto-MCI). The link colors indicate the interdependency strength (cross-MCI) between variables. The number between the link HMD-SPEI denotes the time lags (unit = 6 growing season periods of maize).

Figure 6a, b, and c not only display a non-linear relationship between EPM on HMD and SPEI on HMD, but also show the existence of the predictable relationship among them. The causal analysis suggested a causal link between excessive water event (EPM) and drought event (SPEI), meanwhile heatwave (HMD) became a causal parent for drought event (Fig. 6d). Results further imply that there was no existence of causal connection between heatwave and extreme precipitation.

## Discussion

Results showed strong evidence that South Africa was experiencing the fluctuation of heatwaves during the maize growing season between 1986/87 and 2015/16. The strongest recorded HMD (value of 18) for South Africa was found for the 1994/95 season, followed by 1987/88, 1990/91, 1992/93, 2012/13, and 2015/16 seasons. During these seasons, there was also a soil moisture shortage measured across South Africa^73–75^. The pronounced extreme heatwave thus intensified drought events^76,77^. The finding that the highest values of the 90th percentile of maximum daily temperatures during the last decades were located in northwestern regions is supported by Mbokodo et al.^78^. They concluded that the northwestern region of South Africa had an average maximum temperature above 36 °C from December to February in 1983–2012. The tendency upward trend of heatwaves during the growing season, especially in Free State, North West, and Others regions (Fig. 2) provided further evidence that shifting to maize varieties with higher heat tolerance should be considered essential towards adaptation measures in the future^79,80^. Otherwise, especially under ongoing climate change, the increase in intensity and frequency of heatwaves could shake food security not only for South Africa but also for countries, such as Japan, Taiwan, China, the Republic of Korea, Zimbabwe, and Vietnam, where their domestic needs are depending on South Africa maize exports^81^.

The SPEI in Free State, KwaZulu-Natal, and Mpumalanga did not significantly decrease over time. However, overall, patterns indicate a downward tendency, indicating that drought severity during maize growing season became more pronounced. This pattern is consistent with previous study based on weather station data for Free State and North West^75^. Furthermore, at the end of the study period (2015/16), the degree of drought severity was recorded at the lowest point. According to Climate Change Knowledge Portal (CCKP), the year 2015 for South Africa was the worst drought in century^82^. This implies that drought was one of the primary drivers for maize yield losses during the last 30 years in South Africa^83^. The significant monotonic downward trend of SPEI for the North West, others regions, and at the national scale provide further evidence that South African maize production is likely to experience pronounced drought stress in the future. The combination between increasing temperatures and consistent drought events highlighted the need for mitigation and adaptation strategies at both farm and government levels to increase the resilience of maize yield losses for future drought^84^.

Our analysis did not confirm any significant trend of EPM in the study areas. Contrastingly, Mason et al.^85^ reported that there was a significant increase in extreme precipitation intensity in South Africa from 1931 to 1990. Discrepancies were related to the use of different time series, data sources (meteorological ground station), reference periods, and annual time windows (growing season data used in this study). Nevertheless, both studies reported that lower precipitation covered northwestern, northern, and northeastern regions. The distinction peak of extreme precipitation in 1995/96 and 1999/00 showed an exceptional temporal pattern in all regions. It was reported that extreme precipitation in 1995/96 caused an extreme flooding which was identified as the highest flood peak of the past century^86^. Similarly, extreme precipitation in 1999/00 occurred due to tropical easterly low-pressure and tropical cyclone Eline had caused severe flooding that destroyed the infrastructure and inundated the cropland^87^. The dominance of extreme precipitation in Free State, KwaZulu-Natal, Mpumalanga, and partly Eastern Cape provinces demonstrated that these regions were prone to flooding and water erosion. As reported by Ebhuoma et al.^88^, it was estimated that 35% of the upper uThukela catchment in KwaZulu-Natal was at risk to soil degradation caused by water. Besides the degree of slope and vegetation cover, the precipitation in KwaZulu-Natal influenced soil erosion with a weight factor of 18%. Excessive water could remove the top soil, degrade the soil structure and initiate excess water stress during growing stages which often results in low maize productivity^29,89^. This finding showed that extreme precipitation variability could become a threat to maize production when the water management in crop production is neglected.

The inter-seasonal maize yield in South Africa explained by CSIm at 25% was lower compared to 49% on the global scale^22,23^. The CSIm reveals that maize yield in South African regions was vulnerable to soil moisture deficit. Due to the fact that the irrigation of maize fields in South African regions mainly depends on precipitation, the deficit of water availability event could impact maize yield significantly^52^. This finding showed that the negative yield anomalies in Free State were characterized by the present of extreme heatwaves. Meanwhile in North West, the positive yield anomalies were characterized by the cumulative absence of drought events and extreme precipitation. No significant influences of climate extreme events in KwaZulu-Natal and Mpumalanga indicate that rather non-climatic factors, such as technology improvement, which was not captured by the model, might have been dominant drivers of yield variability. This was in line with our prior study where the agrometeorological indicators, such as temperature, precipitation, solar radiation, and wind speed, showed no correlation with maize yield in KwaZulu-Natal. In contrast with Mpumalanga, only precipitation and wind speed were correlated with maize yield^90^.

It is worth noting that maize yield variability at national scale was not directly influenced by heat stress. Based on the results from causal network analysis, HMD was the casual parent of SPEI which indicated the drought severity during the maize growing season was due to the effect of heatwave intensification. This means that extreme heatwave did not have a direct impact on maize yield at national scale. Exemplarily, Lobell et al.^91^ that maize yield reduction in the United Sates was directly affected by vapor pressure deficit and moisture stress rather than heat stress (temperature above 30 °C). The continuous heatwaves can further dry out the maize plants and the surrounding vegetation, which decreases the evapotranspiration, as a result, the likelihood of precipitation is reduced^92^. Not to mention that this type of causality may occur in the opposite feedback especially in mid latitude regions, when the constant limited water availability lead to a decrease in latent heating (evapotranspiration) and a further increase in sensible heating (surface temperature)^93^.

For a quantitative interpretation of the CSIm data, the yield anomalies were derived from removing the trend that was affected by improvement in the agriculture practice. The novel CSIm was able to improve in capturing the maize yield variability over the standard CSI where extreme precipitation had a significant impact on maize yield variability. Considering that the North West province is one of the main maize production regions and the finding on the upward tendency of extreme heatwave (Fig. 2) which significantly elevates extreme drought events (Figs. 3 and 6), the significant influence of drought event and extreme precipitation event on maize yield (Table 1), the stability of maize production and food security in South Africa could be jeopardized in the future.

In this study, the CSIm model was developed based on historical maize yield and climate data recorded between 1986/87 and 2015/16. Thus, a generalization of our results across time is restricted. For different periods, the model would need to be recalibrated. The reliability of the model capturing the yield variability was fully dependent on the reported maize yield and predictor variables. Thus, the quantitative information on the modified Combined Stress Index must be treated with caution. Despite these restrictions, the finding can be used as a baseline study for future analyses, such as investigating maize yield under predicted climate extreme events.

## Conclusions

The characteristics of extreme heat, drought and precipitation events in South Africa between 1986/87 and 2015/16, i.e., their temporal patterns, trends, frequencies and magnitudes, differed between provinces, suggesting that future adaptation and mitigation strategies should account for spatial differences in their impact. Heatwave and extreme precipitation severity did not significantly increase or decrease over time, however for North West, others regions, and at nationwide, drought severity increased. At the national level maize yield variability was mainly associated with drought events, explaining 25% of the total variability. Maize yield variability in KwaZulu-Natal and Mpumalanga were not influenced by climate extremes. Quantitative results on the impacts of climate extremes are only valid for the observation period. The contemporaneous nonlinear causality results suggests that extreme drought events during the maize growing season were initiated by extreme temperatures above the threshold.

The results highlighted the importance of extreme precipitation in investigating the influence of extreme indices with regard to maize yield variability, particularly in regions that are vulnerable to soil erosion and depending on rainfed irrigation. With the extreme climate events becoming more pronounced in the future, extreme precipitation needs to be included into the model in investigating climate change impacts on yield anomalies.

## Supplementary Information

Supplementary Information.

## Data Availability

The climate data (AgERA5) is openly available in the Copernicus portal (https://cds.climate.copernicus.eu/). The SPEI data set is available through the Global SPEI database (https://spei.csic.es/index.html).
