# Supplementary material for: Impact of climate extreme events and their causality on maize yield in South Africa
Source: Sci Rep. 2023 Aug 1;13:12462. doi: 10.1038/s41598-023-38921-0 (PMC10393995; doi:10.1038/s41598-023-38921-0)
Supplement: Supplementary file 1 — Supplementary Information. [file 41598_2023_38921_MOESM1_ESM.docx]

**Impact of climate extreme events and their causality on maize yield in South Africa**

**Christian Simanjuntak^1,^*, Thomas Gaiser^1^, Hella Ellen Ahrends^2^,** **Andrej Ceglar^3^, Manmeet Singh^4^,** **Frank Ewert ^1,5^, and Amit Kumar Srivastava^1^**

^1^Institute of Crop Science and Resource Conservation, University of Bonn, Katzenburgweg 5, 53115, Bonn, Germany.

^2^Department of Agricultural Sciences, University of Helsinki, Koetilantie 5, 00014, Helsinki, Finland.

^3^Climate Change Centre of the European Central Bank, Sonnemannstrasse 20, 60314, Frankfurt am Main, Germany.

^4^Indian Institute of Tropical Meteorology, Ministry of Earth Sciences, Pune, India.

^5^Leibniz Centre for Agricultural Landscape Research (ZALF), Eberswalder Straße 84, 15374, Müncheberg, Germany.

*Email: simanjuntak_christ@yahoo.co.id

**Supplementary materials**

South Africa irrigated and rainfed cropland


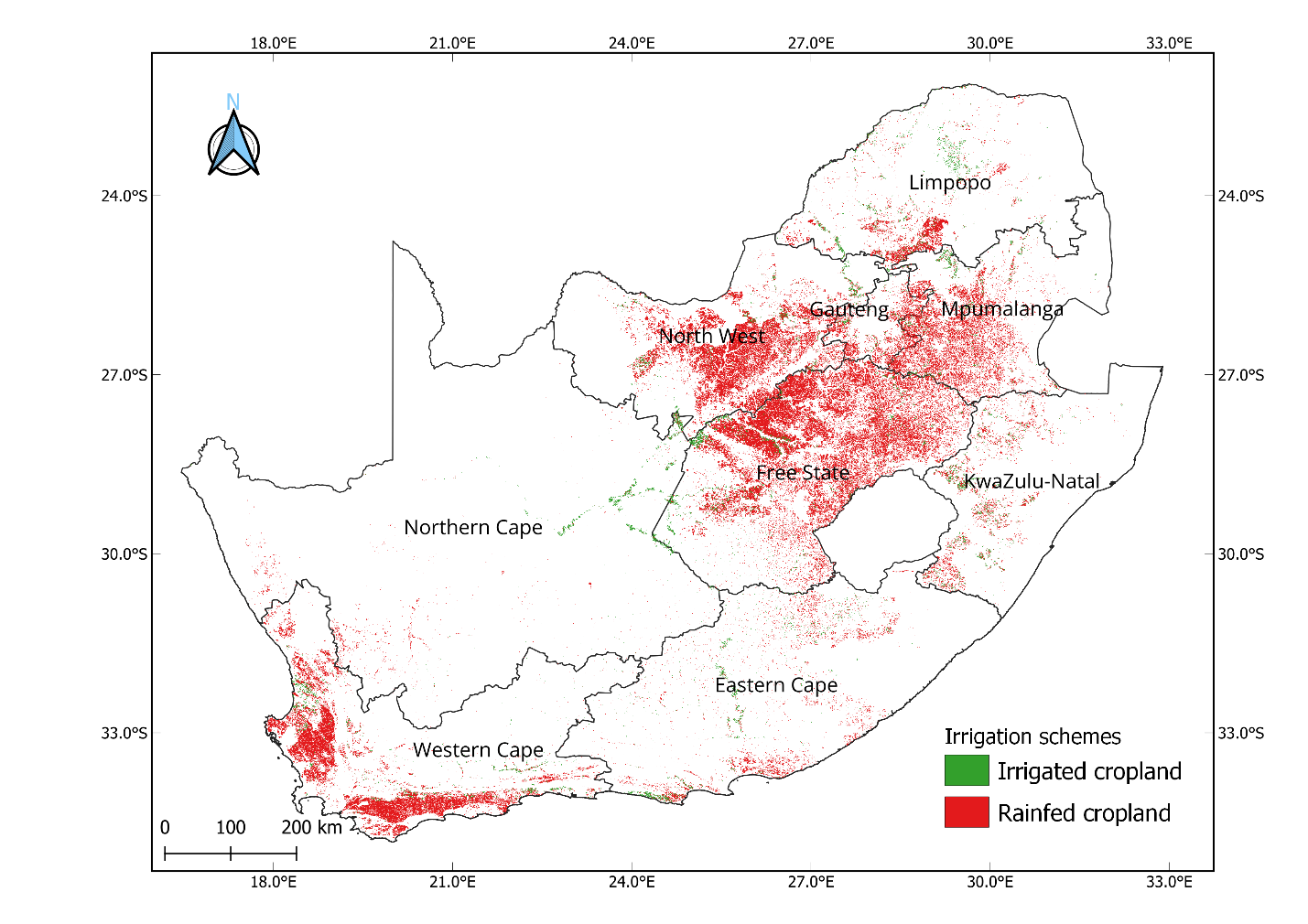


**Figure S1**. Irrigated and rainfed cropland in South Africa. The map was generated from South African National Land-Cover 2018 dataset at 20-meter resolution using QGIS 3.16.7-Hannover, software, https://www.qgis.org/en/.

South Africa Map


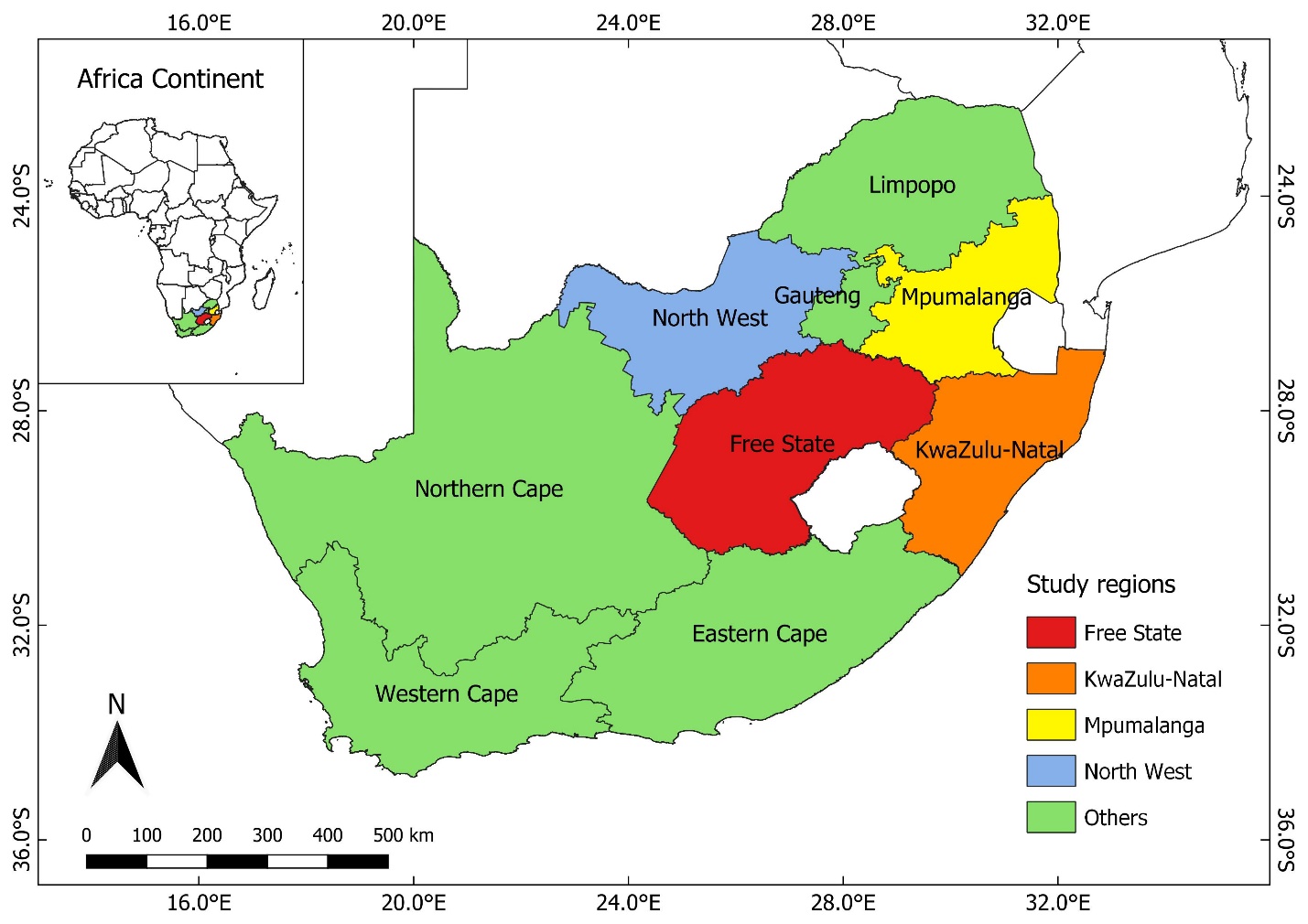


**Figure S2**. The map of 6 regions used in this study, namely Free State, KwaZulu-Natal, Mpumalanga, North West, Other regions (combination of Limpopo, Gauteng, Northern Cape, Eastern Cape, and Western Cape), and the entire regions of South Africa. The maps were created using QGIS 3.16.7-Hannover, software, https://www.qgis.org/en/.

Multicollinearity analysis output prior to multiple linear regression analysis

**Table S1**. Multicollinearity analysis for explanatory variables.

| **Regions** | **Variables** | **Tolerance** | **VIF** |
| --- | --- | --- | --- |
| Free State | HMD | 0.986 | 1.013 |
|  | SPEI | 0.578 | 1.728 |
|  | EPM | 0.575 | 1.736 |
| KwaZulu-Natal | HMD | 0.882 | 1.133 |
|  | SPEI | 0.684 | 1.460 |
|  | EPM | 0.618 | 1.617 |
| Mpumalanga | HMD | 0.901 | 1.108 |
|  | SPEI | 0.792 | 1.262 |
|  | EPM | 0.775 | 1.289 |
| North West | HMD | 0.944 | 1.059 |
|  | SPEI | 0.798 | 1.253 |
|  | EPM | 0.762 | 1.312 |
| Others | HMD | 0.981 | 1.019 |
|  | SPEI | 0.805 | 1.241 |
|  | EPM | 0.795 | 1.257 |
| South Africa | HMD | 0.952 | 1.051 |
|  | SPEI | 0.894 | 1.118 |
|  | EPM | 0.928 | 1.076 |

Multicollinearity analysis output prior to multiple linear regression analysis

**Table S2**. Multicollinearity analysis between explanatory variables.

| **Regions** | **Condition index** | **Intercept** | **HMD** | **SPEI** | **EPM** |
| --- | --- | --- | --- | --- | --- |
| Free State | 1.00 | 0.00 | 0.02 | 0.16 | 0.16 |
|  | 1.29 | 1.00 | 0.00 | 0.00 | 0.00 |
|  | 1.31 | 0.00 | 0.97 | 0.01 | 0.01 |
|  | 2.18 | 0.00 | 0.00 | 0.82 | 0.82 |
| KwaZulu-Natal | 1.00 | 0.00 | 0.08 | 0.15 | 0.17 |
|  | 1.29 | 1.00 | 0.00 | 0.00 | 0.00 |
|  | 1.32 | 0.00 | 0.70 | 0.17 | 0.00 |
|  | 2.06 | 0.00 | 0.21 | 0.66 | 0.82 |
| Mpumalanga | 1.00 | 0.00 | 0.13 | 0.17 | 0.18 |
|  | 1.28 | 1.00 | 0.00 | 0.00 | 0.00 |
|  | 1.44 | 0.00 | 0.85 | 0.17 | 0.07 |
|  | 1.71 | 0.00 | 0.01 | 0.64 | 0.74 |
| North West | 1.00 | 0.00 | 0.09 | 0.20 | 0.22 |
|  | 1.24 | 0.09 | 0.00 | 0.00 | 0.00 |
|  | 1.29 | 0.00 | 0.81 | 0.16 | 0.01 |
|  | 1.71 | 0.00 | 0.09 | 0.63 | 0.76 |
| Others | 1.00 | 0.00 | 0.05 | 0.24 | 0.25 |
|  | 1.21 | 1.00 | 0.00 | 0.00 | 0.00 |
|  | 1.24 | 0.00 | 0.92 | 0.06 | 0.01 |
|  | 1.63 | 0.00 | 0.01 | 0.69 | 0.73 |
| South Africa | 1.00 | 0.00 | 0.17 | 0.27 | 0.21 |
|  | 1.18 | 1.00 | 0.00 | 0.00 | 0.00 |
|  | 1.24 | 0.00 | 0.64 | 0.00 | 0.39 |
|  | 1.41 | 0.00 | 0.18 | 0.72 | 0.39 |
